# Reduced Set of Virulence Genes Allows High Accuracy Prediction of Bacterial Pathogenicity in Humans

**DOI:** 10.1371/journal.pone.0042144

**Published:** 2012-08-06

**Authors:** Gregorio Iraola, Gustavo Vazquez, Lucía Spangenberg, Hugo Naya

**Affiliations:** 1 Unidad de Bioinformática, Institut Pasteur Montevideo, Montevideo, Uruguay; 2 Sección Genética Evolutiva, Facultad de Ciencias, Universidad de la República, Montevideo, Uruguay; 3 Departamento de Ciencias e Ingeniería de la Computación, Universidad Nacional del Sur, Bahía Blanca, Argentina; 4 Departamento de Producción Animal y Pasturas, Facultad de Agronomía, Universidad de la República, Montevideo, Uruguay; Cairo University, Egypt

## Abstract

Although there have been great advances in understanding bacterial pathogenesis, there is still a lack of integrative information about what makes a bacterium a human pathogen. The advent of high-throughput sequencing technologies has dramatically increased the amount of completed bacterial genomes, for both known human pathogenic and non-pathogenic strains; this information is now available to investigate genetic features that determine pathogenic phenotypes in bacteria. In this work we determined presence/absence patterns of 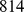 different virulence-related genes among more than 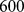 finished bacterial genomes from both human pathogenic and non-pathogenic strains, belonging to different taxonomic groups (i.e: *Actinobacteria*, *Gammaproteobacteria*, *Firmicutes*, etc.). An accuracy of 95% using a cross-fold validation scheme with in-fold feature selection is obtained when classifying human pathogens and non-pathogens. A reduced subset of highly informative genes (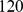) is presented and applied to an external validation set. The statistical model was implemented in the BacFier v1.0 software (freely available at 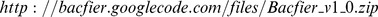), that displays not only the prediction (pathogen/non-pathogen) and an associated probability for pathogenicity, but also the presence/absence vector for the analyzed genes, so it is possible to decipher the subset of virulence genes responsible for the classification on the analyzed genome. Furthermore, we discuss the biological relevance for bacterial pathogenesis of the core set of genes, corresponding to eight functional categories, all with evident and documented association with the phenotypes of interest. Also, we analyze which functional categories of virulence genes were more distinctive for pathogenicity in each taxonomic group, which seems to be a completely new kind of information and could lead to important evolutionary conclusions.

## Introduction

Several factors, including globalization and sanitation conditions, have been shaping the world’s landscape of infectious diseases over the years. In developed countries, 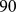 percent of documented infections in hospitalized patients are caused by bacteria. These cases probably show only a small proportion of the actual number of bacterial infections occurring in the entire population, and they usually represent the most severe cases. In developing countries, a variety of bacterial infections often provoke a devastating effect on the inhabitants’ health. The World Health Organization (WHO) has estimated that each year, 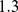 million people die of tuberculosis, 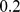 million die of pertussis and 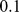 million die of syphilis. Diarrheal diseases, many of which are of bacterial etiology, are the second leading cause of death in the world (after cardiovascular diseases), killing 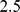 million people annually (WHO, 2008). This scenario evidences that even today, infectious diseases are a permanent threat for human health around the world.

Understanding the biology of the causative agents of these diseases has been a permanent challenge since the beginning of bacteriology. Nowadays, the mechanisms involved in the virulence (defined as the relative capacity of a microbe to cause damage in a host) of pathogenic bacteria are widely studied in clinical bacteriology, but the advent of new technologies has enabled their study from different perspectives. In this context, bacterial genomics have greatly contributed to the better understanding of pathogenicity due to the possibility of generating and comparing whole genome sequences. The onset of this discipline started with the automation of Sanger sequencing chemistry and the completion of *Haemophilus influenzae* and *Mycoplasma genitallium* genomes [1], [2] in the mid-1990 s; since then, projects to sequence the genomes of a large number of organisms were undertaken by means of this method [3]–[5]. However, during the last decade, to cover the increasing sequencing demands, new non-Sanger high-throughput sequencing systems have been developed under the name of “second generation” or “next-generation” sequencing technologies [6], [7]. These developments have significantly reduced the cost and simultaneously increased the speed of DNA sequencing. In this sense, the great majority of organisms whose genomes have been sequenced so far are bacteria, with 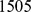 complete and published genome sequences and 6037 ongoing projects (http://www.genomesonline.org/cgi-bin/GOLD/bin/gold.cgi).

Comparative genomics, including comparison at the DNA, transcriptome, and proteome levels, have emerged as a key to give a biological sense to all this massive information. Focused on improving the knowledge on pathogenicity determinants two bioinformatic approaches have been used, based on two complementary explanations for bacterial pathogenesis. On the one hand, pathogenicity has been related to amino acid substitutions which lead to modified protein structures, and probably modified functions [8]–[10]. In this case, a particular gene shared by a human pathogenic species and a non-pathogenic species, could be causing a pathogenic phenotype in the first one, determined by non-synonymous mutations that modify key aminoacids and alter protein function. Based on this, our group has recently published a method that detects variable regions inside protein sequences which can be potentially related to pathogenicity [11].

On the other hand, trying to give an integrative view of bacterial pathogenicity prediction from a bioinformatic’s perspective, in this work we exploit an alternative explanation for bacterial pathogenicity. Pathogenicity has been attributed to the presence or absence of genes which confer particular pathogenic phenotypes, like toxins [12]. In this case, these genes would be present in pathogenic species but absent in non-pathogenic ones. The most widely spread approach to evaluate this is the pairwise comparison between genomes of pathogenic and non-pathogenic bacteria or even multiple comparisons between different strains of the same species [13]–[15]. These kinds of approaches can give information regarding the presence or absence of genes involved in pathogenicity of a particular species or even a genus. However, it is difficult to extrapolate this information to higher taxonomic levels, which keeps us from drawing conclusions about general features that are determining bacterial pathogenicity.

For this reason, our motivation was: i) try to identify presence/absence patterns of virulence-related genes which could explain the pathogenic phenotype of bacteria at higher taxonomic levels than species or genus, ii) discuss the biological significance of those genes giving an integrative view of genetic determinants of bacterial pathogenicity, iii) use this information to develop a machine learning model to classify bacterial genomes into human pathogens and non-pathogens and iv) implement this model in a software that can be used to predict pathogenicity in the upcoming sequenced bacterial genomes. The last two points are particularly interesting because a statistical model implemented in an easy-to-use software, capable of predicting bacterial pathogenicity based on genomic information, can be helpful for practical purposes. For example, in food or pharmaceutical industries it is essential to know the pathogenic potential of bacterial strains used in bioengineering.

## Results and Discussion

The idea that bacterial species can be effectively grouped into human pathogens and non-pathogens based on their virulence-genes composition, arises from preliminary results that indicated differential patterns in presence or absence of these kind of genes among both groups (human pathogens and non-pathogens).

**Figure 1:**
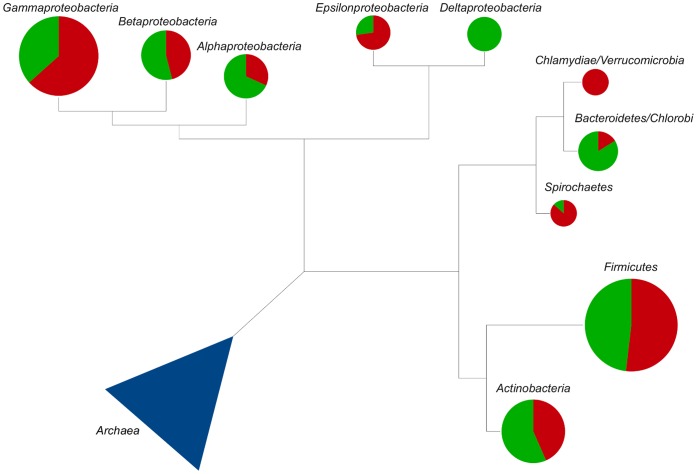
Phylogenetic relations of bacterial groups used in this work. Chart sizes are proportional to the number of genomes present in each taxonomic group. The precentage of pathogenic organisms is shown in red and green is used for non-pathogenic.

All finished and annotated genomes of human pathogenic and non-pathogenic bacteria were used to perform a presence/absence analysis over 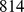 groups of orthologous genes belonging to 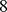 functional categories (toxins, two-component systems, ABC transporters, motility, flagellar assembly, LPS biosynthesis, secretion systems and chemotaxis), in order to determine which ones are strongly related to pathogenicity in different bacterial taxonomic groups (*Actinobacteria*, *Alphaproteobacteria*, *Betaproteobacteria*, *Bacteroidetes*/*Chlorobi*, *Chlamydiae*/*Verrucomicrobia*, *Deltaproteobacteria*, *Epsilonproteobacteria*, *Firmicutes*, *Gammaproteobacteria*, *Spirochaetes*, etc.). Figure 1 shows phylogenetic relations and the proportion of pathogenic and non-pathogenic organisms in studied taxa.

**Figure 2:**
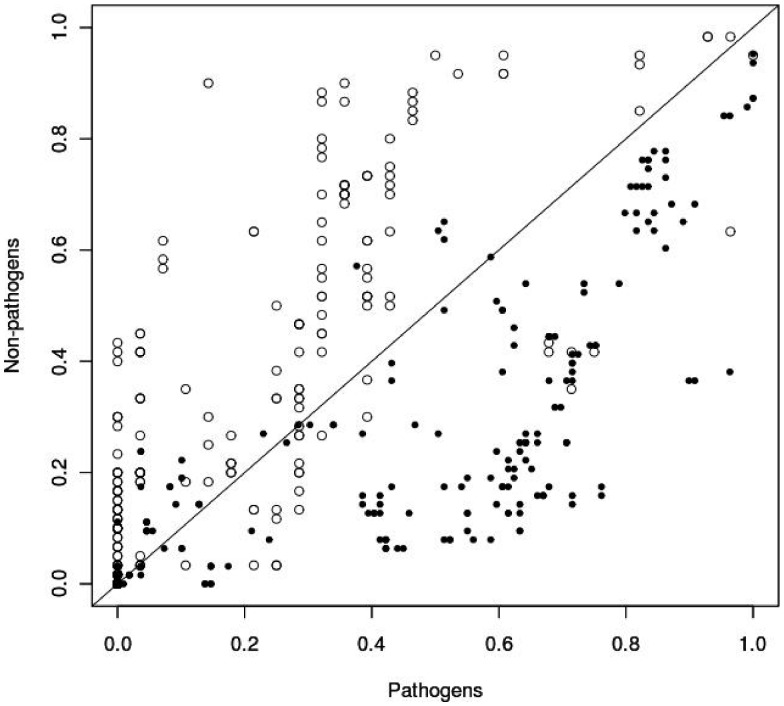
Frequency distribution of ABC transporter genes in *Alphaproteobacteria* and *Gammaproteobacteria*. For each gene, abcisse value is the number of pathogenic strains inside a certain taxonomic group in which it is present, divided by the total number of pathogenic strains inside the taxonomic group. The ordinate value is the same but for the non-pathogenic strains inside the group. White circles show that genes coding for ABC transporters are more frequent in pathogenic species of *Gammaproteobacteria* than in non-pathogenic species of this group. The opposite pattern is observed for *Alphaproteobacteria* in black circles.

The analysis was accomplished by calculating the frequency of genes belonging to each functional category in pathogenic and non-pathogenic species of each taxon. The assumed null hypothesis was that, if a certain gene is not related to pathogenicity, its frequency would not be biased towards pathogenic or non-pathogenic species; furthermore, it would be almost equally distributed within both classes. Genes presenting a high frequency among pathogens and a low frequency in non-pathogens are probably contributing to a pathogen-related phenotype, for example genes coding for toxins. Conversely, a gene that presents low frequency among pathogens and high frequency in non-pathogens could be indicating the loss of genes coding for redundant functions. For example, proteins that transport certain molecules across membranes, which are essential for a free-living style, are often dispensable when bacteria are well-adapted to the environment inside their hosts. The frequency distribution of ABC transporter genes in *Alphaproteobacteria* and *Gammaproteobacteria* clearly exemplifies this situation. Figure 2 shows the frequency of each gene in pathogenic and non-pathogenic organisms. Points falling on the diagonal line represent genes whose frequency is balanced between pathogens and non-pathogens. Points closer to the Y axis are more represented in non-pathogens and points closer to the X axis are more frequent in pathogens. As it is shown in this figure, ABC genes are strongly related to non-pathogenic species in *Alphaproteobacteria*, while there are overrepresented in pathogenic species in *Gammaproteobacteria* (Figure 2).

**Figure 3:**
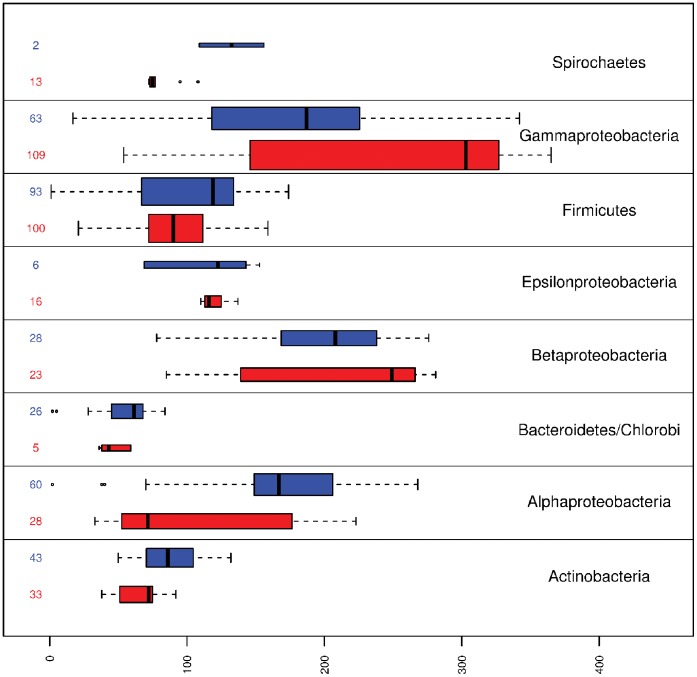
Boxplot representing the presence of genes per taxonomic group. The length of each box represent the number of genes present in both pathogenic (red) and non-pathogenic (blue) organisms for each taxonomic group considered. The number of organisms inside each group are shown leftside, this number is proportional to box width. Dark vertical lines show the median for the amount of present genes per group, box limits represent quartiles and whiskers extend to the most extreme data point which is no more than 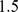 times the interquartile range.

**Figure 4:**
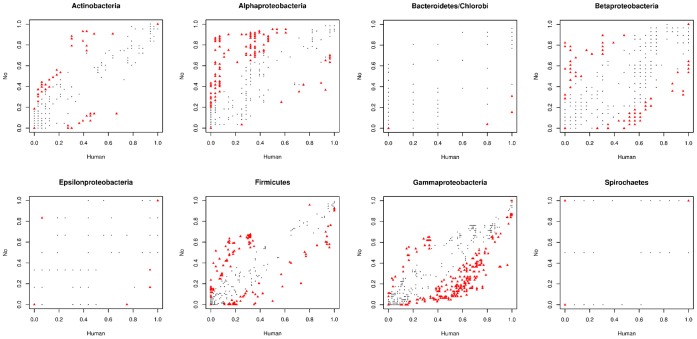
Frequencies of each of 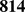 genes per bacterial taxonomic group. Frquency calculation was performed for each gene as in Figure 2. Red triangles show significative genes that apart from the null distribution (same frequency in pathogens and non-pathogens) by exact Fisher test, black circles are non significative genes.

As shown in Figure 3 the number of present genes is highly variable among classes (pathogens and non-pathogens) and even between taxonomic groups. Moreover, a great number of these present genes, belonging to the 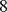 functional categories, presented a frequency bias towards either pathogenic or non-pathogenic species (Figure 4), deviating from the proposed null hypothesis. These findings supported the idea that presence/absence patterns of virulence-related genes are informative enough to discriminate between human pathogenic and non-pathogenic bacterial species (Table 1), indicating that this data can be used to construct a classification model based on highly significant biological information.

**Table 1:** Statistical overview of data distribution among taxonomic groups.

			Class NP					Class HP			
Taxon	Purpose^1^	n	median	IQR	min	max	n	median	IQR	min	max
*Actinobacteria*	M	43	17.0	7.00	9	28	33	12.0	4.00	5	20
*Alphaproteobacteria*	M	60	28.5	13.50	0	49	28	10.0	23.00	5	37
*Bacteroidetes*/*Chlorobi*	M	26	10.5	3.75	0	15	5	8.0	4.00	7	11
*Betaproteobacteria*	M	28	29.5	11.50	11	47	23	39.0	25.00	14	49
*Epsilonproteobacteria*	M	6	17.5	9.75	7	20	16	14.0	0.25	13	20
*Firmicutes*	M	93	20.0	10.00	0	30	100	16.0	10.00	3	30
*Gammaproteobacteria*	M	63	25.0	15.00	1	47	109	43.0	24.00	9	51
*Spirochaetes*	M	2	20.0	6.00	14	26	13	9.0	1.00	8	14
*Chlamydiae*/*Verrucomicrobia*	T	–	–	–	–	–	14	11.0	0.00	10	12
*Deltaproteobacteria*	T	28	22.0	5.25	6	31	–	–	–	–	–

Statistical variation is measured as the interquartile range (IQR) in human pathogens (HP) and non-pathogens (NP).

1 M: used in model construction and testing, T: used only in model testing.

### Classification Model

We used a machine learning approach based on a cross-validation scheme with in-fold feature selection together with a linear Support Vector Machine (SVM) classifier. Preliminary models were constructed using the whole 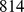 set of genes, but the number of genes was systematically reduced by means of a feature selection process. The definitive model included the first 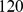 genes ranked by their significance for classification (Table S1). However, since the number of variables is still high, problems associated with chance correlation might arise. For these reason a y-randomization test was implemented. Figure S1 shows the performance obtained in the test (50% accuracy), indicating the absence of chance correlation. Section Model construction further explains these methodologies.

The number of correctly/incorrectly classified genomes in the complete set was 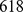/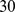, obtaining an accuracy of 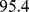
% (Table S2). Table 2 describes the classification performance related to all bacteria taxonomy considered in the dataset. The last column of the table indicates the classification success rate for each group considered in the taxonomy; all values were obtained using the 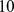-fold cross validated SVM model, not by retraining the model using only organisms of the particular taxon. The performance is preserved across the whole taxonomy, ranging from 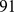
% in *Epsilonproteobacteria*, up to 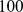
% in *Bacteroidetes*/*Chlorobi*. Mid-size groups like *Betaproteobacteria*, *Actinobacteria* and *Alphaproteobacteria* showed a prediction success rate similar or better than the general performance rate. Finally the *Firmicutes*, the biggest group, showed an excellent classification level of 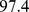
%. Classification performance according to class labels is shown in Table 3, the general error rate is almost equal for false positives and negatives and the general success rate is also equal for pathogens and non-pathogens.

**Table 2:** Classication performance for each taxonomic groups used to construct the model.

		Class NP		Class HP		
	Number	Predicted as NP	Predicted as HP	Predicted as NP	Predicted as HP	correct classif. rate
*Actinobacteria*	76	42	1	32	1	97.4%
*Alphaproteobacteria*	88	54	6	28	0	93.2%
*Bacteroidetes*/*Chlorobi*	31	26	0	5	0	100%
*Betaproteobacteria*	51	27	1	23	0	98.1%
*Epsilonproteobacteria*	22	6	0	14	2	91%
*Firmicutes*	193	91	2	97	3	97.4%
*Gammaproteobacteria*	172	59	4	107	2	96.5%
*Spirochaetes*	15	2	0	13	0	100%

Inside each class the number of correct and incorrect classified genomes are shown.

**Table 3:** Confusion matrix showing average classification performance across all taxonomic groups.

Classified as	Pathogenic	Non-pathogenic
Pathogenic	313 (95.2%)	15 (4.8%)
Non-pathogenic	15 (4.9%)	308 (95.1%)

### Model Testing and Comparison

To further test the SVM model we evaluated its performance by analyzing genomes originally not included in the dataset used to construct the model. On the one hand, we defined a Group I of 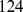 genomes with known labels for human pathogen or non-pathogen, originally excluded from the dataset due to reduced number of genomes per group or misrepresentation of one of the two classes. On the other hand, we defined a Group II of 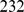 “blind” genomes without previous information for pathogenicity.

Group I genomes were classified with an accuracy of 98% (Table 4), even better than the average 95.4% obtained during cross-validation procedure using the original dataset. Only in two taxonomic groups (*Chlamydiae*/*Verrucomicrobia* and *Fusobacteria*) the model showed an accuracy lower than 100%, and in each case only one genome was misclassified. Group II genomes were previously subjected to an exahustive bibliographic search in order to assign them to human pathogens or non-pathogens (Table S3). Application of SVM model over this group resulted in 92% of average accuracy (Table 4), ranging from 87% in *Epsilonproteobacteria* to 100% in *Deltaproteobacteria*, *Bacteroidetes*, etc. The fact that accuracy is preserved in both test groups reaffirms the results obtained when performing the cross-validation scheme, indicating that our model is robust and the high performance in classification and prediction of human pathogens and non-pathogens is independent of the dataset used to build the model.

**Table 4:** Classification performance for Group I and Group II.

	Taxon	Correctly classified	Wrongly classified	Accuracy
	*Chlamydiae*	14	0	100%
	*Deltaproteobacteria*	26	0	100%
	*Planctomycetes*	3	0	100%
	*Deinococcus-Thermus*	3	0	100%
	*Acidobacteria*	3	0	100%
Group I	*Deltaproteobacteria*	4	1	80%
	*Chloroflexi*	8	0	100%
	*Cyanobacteria*	27	1	96.4%
	*Thermotogae*	9	0	100%
				
	*Other bacteria*	19	0	100%
	*Actinobacteria*	26	4	87%
	*Alphaproteobacteria*	24	2	92%
	*Bacteroidetes*	13	0	100%
	*Betaproteobacteria*	22	2	91%
	*Deltaproteobacteria*	5	0	100%
Group II	*Epsilonproteobacteria*	8	1	89%
	*Firmicutes*	42	4	91%
	*Gammaproteobacteria*	38	4	90.5%
	*Chloroflexi*	6	0	100%
	*Cyanobacteria*	11	1	91%
	*Deinococcus-Thermus*	7	0	100%
	*Other bacteria*	13	0	100%

The SVM model was also compared to a method developed by Andreatta et al. [16], which is the unique tool reported so far with the same purpose of predicting bacterial pathogenicity. Andreatta et al. proposed a classifier for the prediction of pathogenicity restricted only to *Gammaproteobacteria*, considering a dataset of 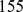 organisms and obtaining an accuracy of 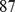
%. This is lower than the 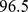
% achieved for the same taxonomic group (using 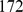 organisms) with our SVM model, and even worse than the general performance of our classifier (95.4%). Furthermore, in the particular case of *Gammaproteobacteria*, our method presented a lower error rate in misclassifying human pathogens as non-pathogens (only 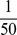), than the other way around (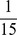 non-pathogens classified as pathogens). This is of crucial importance in practical applications (such as for clinical or industrial purposes), since the social costs of misclassifying a pathogenic strain as non-pathogenic are usually higher than the opposite scenario.

### Biological Interpretation

The eight pathogenicity-related functional categories investigated in this work were represented in the set of 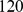 genes selected for the classifier. Forty genes belonged to ABC transporters, 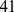 corresponded to two-component systems and chemotaxis proteins, 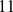 corresponded to toxins, 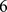 belonged to the LPS biosynthesis pathway and 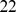 coded for flagellar assembly proteins, motility proteins and proteins from secretion systems. We selected from each group the most distinctive genes and discussed their biological meaning considering their implications in bacterial pathogenesis (Table 5).

**Table 5:** Summary of the biological relevance for pathogenicity of a reduced subset of the selected 120 genes.

Functional category	Genes	Comment
ABC	*sitC*, *hrtB*, *btuD*, *gluD*	Strong association between pathogens and the presence of transporters for metallic cations, vitamin B12, phosphate and amino acids
TCS&CH	*vicK*, *qseC*	VicK absent in most non-pathogenic *Firmicutes*. QseC is present in most pathogenic *Gammaproteobacteria*, but absent in *Yersinia*
LPS	*lpxK*, *wapR*, *rgpA*, *rfbP*	Genes involved in LPS biosynthesis did not show differences in presence/absence patterns between pathogens and non-pathogens
FLA&MOT	*flbP*, *fimH*, *fimI*, *pilA*	FlbP is found in pathogenic *Spirochaetes*. FimH and FimI are found in *Enterobacteraceae*. PilA is present in pathogens of a group of families inside *Gammaproteobacteria*
SS	*tatA*, *yscC*, *ppkA*	TatA is found in pathogenic *Epsilonproteobacteria*. YscC is part of T3SS from *Y. pestis* and many other pathogens. PpkA is part of T6SS from *Pseudomonas*
TOX	*slo*, *tlh*, *cdtC*	SLO is present in more than 20 pathogenic Gram-positive bacteria, including Firmicutes. Thermolabile hemolysin is exclusive from Vibrio. CdtC is present in a wide broad of pathogens including *Campylobacter*

The functional categories are described in Methods section.

#### ABC transporters

ABC transporters are specialized proteins that function as either importers, which bring nutrients and other molecules into cells, or as exporters, which pump toxins, drugs and lipids across membranes [17]. Based on the kind of substrate ABC transporters are specific for: i) metallic cations, iron-siderophore and vitamin B12, ii) phosphate and amino acids, iii) oligosaccharides and polyol, iv) monosaccharides, v) mineral and organic ions, vi) peptides and nickel and vii) others (ABC-2). Our classification model selected those ABC transporters related to transport of metallic cations, vitamin B12, phosphate and amino acids as the most important.

It is widely known that metallic ions, are essential for prokaryotic cell physiology. The amount of these ions is not constant inside the hosts of pathogenic bacteria, and their concentration is sometimes considerably lower than needed [18]. The presence of systems implied in metallic cations scavenging is mandatory for bacterial survival inside host cells, and it is a key feature for downstream processes like the development of pathogenic phenotypes [19].

The emergence of most pathogenic species is associated with an evolutionary transition from a free-living to a host dependent lifestyle, to a certain extent. Bacterial genomes, and especially those from pathogens, abide by the maxim “use it or leave it”, where genes or even whole gene pathways are lost if their products are not essential for cell maintenance, or can be taken from the environment [20]. Two examples are amino acid and vitamin biosynthesis pathways, which have been lost in most pathogens [21]. In this sense, the high representation of these types of ABC systems support the idea that it is more convenient for pathogens to incorporate these compounds from the host environment than to produce them *de novo*.

#### Two component systems and chemotaxis

Two-component systems (TCS) are widespread signal transduction pathways among bacteria, which play a crucial role in adaptation to fluctuating surroundings by sensing changes in environmental conditions [21], like those experimented during process of entry, colonization and spread [21]. Genes belonging to 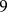 TCS families were selected by the classifier as most informative, being OmpR and NtrC the families with the highest TCS representation.

Osmolarity sensors EnvZ-OmpR and CpxA-CpxR (OmpR family) regulate the expression of outer membrane porins in Gram-negative bacteria. Porins control osmolar pressure in response to environmental changes, like from a free-living context to inside a host cell [22].

Gene *vicK* is part of *Bacillus subtilis* VicR-VicK system (also a member of OmpR family). It has been widely related to exopolysaccharide biosynthesis, biofilm formation and virulence factors expression in Gram-positives [23], [24]. Gene *vicK* is absent in an important group of non-pathogenic *Firmicutes*, including most non-pathogenic species of genus *Clostridium*. Seemingly, this feature allows the correct classification of these species and is also indicating a certain importance of the VicR-VicK system in some point of *Clostridium* pathogenesis.

The QseB-QseC system is involved in regulation of motility proteins [25], which are key virulence factors of many bacterial pathogens. Often, this system has pleiotropic effects over phenotypes including chemotaxis, adherence, host cell invasion, colonization and innate immune signaling [26]. It was identified in most distinctive pathogenic members of *Gammaproteobacteria*, including *Salmonella*, *Escherichia*, *Vibrio*, and *Shigella*. Surprisingly, it was absent in *Yersinia pestis*’ genomes.

Genes representing 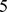 TCS for NtrC family were selected. Among them we found PilS-PilR, another TCS involved in adherence and cell invasion. This system is essential for type IV secretion systems induction in *Neisseriaceae* species, like *Kingella kingae* an increasingly common cause of septic arthritis, bacteremia, and osteomyelitis in young children [27]. Interestingly, orthologous genes of *pilR* were found in a small group of *Gammaproteobacteria*, including *Pseudomonas aeruginosa*, *Acitnetobacter baumanii* and *Legionella pneumophila*.

#### Toxins

Pathogenic bacteria have been developing a variety of strategies to manipulate host cell functions, often involving toxins [12]. These proteins have a wide range of action, causing different effects, like host cells deregulation, protein synthesis interruption or membrane damage [28]–[30]. A total of 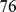 different bacterial toxins were included in this work. Feature selection analysis selected 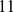 toxins for the model.

Streptolysin O (SLO) is a thiol-activated cytolysin, the effect of this pore-forming toxin is more subtle than simple lysis of host cells, and may include interference with immune cell function [31]. SLO is synthesized by more than 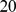 species of Gram-positive bacteria [32], and it is intimately involved in pathogenesis of *Arcanobacterium pyogenes*, *Clostridium perfringens*, *Listeria monocytogenes* and *Streptococcus pneumoniae* [31]. In this work, SLO was identified in pathogenic *Firmicutes* and absent in non-pathogenic species of this group. This gene is present in most pathogenic strains of *S. pyogenes*, *S. pneumoniae* and those species described by Billington et al. [31], but it is also present in pathogenic *Bacillus cereus*, *Streptococcus dysgalactiae* and *Gardnerella vaginalis*, the latter belonging to *Actinobacteria*.

Hemolysin II and thermolabile hemolysin are also pore-forming toxins selected by the model. The first is produced by pathogenic species of genus *Bacillus*, [33], [34] although, in this work, genes extremely similar to hemolysin II were also identified in all pathogenic strains of *Staphylococcus aureus*. Thermolabile hemolysin is characteristic of *Vibrio* species [35] as confirmed by the identification of this gene exclusively in *V. cholerae* and *V. vulnificus* strains.

Cytolethal distending toxin is able to block the host cell cycle between G2 and mitosis [28]. As described in previous works it was identified in a broad range of pathogenic bacteria including *Campylobacter* spp., *Salmonella enterica*, *Haemophilus ducreyi* and *Actinobacillus actinomycetemcomitans* [31]. A/B toxins have similar effects in cell-cycle deregulation, affecting migration, morphogenesis, cell division [36] and membrane trafficking [37]. These were identified in *Clostridium difficile* and in many pathogenic strains of *Escherichia coli*, including O157:H7, O55:H7, O127:H6 and O103:H2. In addition to the contribution for classification, the presence of A/B toxin in these phylogenetically distant groups of possibly indicates horizontal gene transfer events between them.

#### LPS biosynthesis

Lipopolysaccharides (LPS) are major components of the outer membrane of Gram-negative bacteria, which can be recognized by the host’s toll-like receptor 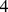 (involved in inflammatory response). High concentrations of LPS can induce fever, increase heart rate, and lead to septic shock and death [38].

The model selected six (*lpxK*, *wapR*, *rgpA*, *gmhB*, *rfe* and *rfbP*) out of 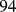 genes, which code for proteins comprising different steps of typical Gram-negative LPS biosynthesis. Tetraacyldisaccharide 4′-kinase (*lpxK*) catalyzes one of the last steps for Lipid A biosynthesis [39]. Genes *wapR* and *rgpA* produce rhamnosyltransferases, which add rhamnose to the polysaccharide backbone. In particular cases, the incorporation of L- or R-rhamnose determines different glycoforms of the core region, leading to LPS variability, hence virulence [40]. Two genes are involved in O-antigen biosynthesis: *rfbP* codes for a glycosyltransferase responsible for the first step in O-antigen biosynthesis [41], while *rfe* (*wecA*) catalyzes the first membrane step of O-antigen and enterobacterial common antigen biosynthesis in *E. coli*. Its involvement in the virulence of Gram-negative bacteria has also been reported [42].

In spite of being selected by the model as relevant for classification, none of these genes showed a clear presence/absence pattern among pathogenic and non-pathogenic species. However, this does not mean they are not informative; on the contrary, these genes may be contributing to classification by an additive effect, being their individual inputs restricted to more particular groups.

#### Flagellar assembly and motility

Bacterial motility is a major factor in pathogenesis. This feature is involved in processes like biofilm formation, host cell colonization and bacterial spread inside the host [43]. Flagellar macromolecular machinery is the paradigm of bacterial motility, being present in a wide range of human pathogens, including *E. coli*, *S. enterica* and *P. aeruginosa* [44]–[46]. In the present work, 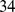 different genes involved in flagellum formation were investigated. Additionally, other 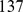 genes involved in different mechanisms related to bacterial motility (fimbrial proteins, adhesins, chemosensory proteins and regulatory proteins) were included.

Five genes directly involved in flagellar biosynthesis (*fliA*, *fliD*, *fliK*, *fliL* and *fliW*) were selected by the model. Gene *fliA* codes for 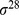, responsible for the regulation of flagellin biosynthesis. Inactivation experiments of *fliA* in *P. aeruginosa* cause non-motility, due to inability of expressing the flagellin gene [47]. The *fliD* gene codes for a structural component of the flagellar cap, which is important in host cell adhesion and colonization [48]. Gene *fliL* is dispensable for swimming in pathogenic species like *E. coli* and *S. enterica* [49], but it is essential for swarming (flagellar-dependent motility in solid medium) in these species. Gene *fliK* is responsible for controlling flagellar hook length, which directly affects the performance of the flagella in producing translational motion [50]. Gene *fliW* codes for a new flagellin assembly protein in *Treponema pallidum* which has orthologous in many related species [51].

Gene *flbB* is part of the flagellar motor exclusively in *Spirochaetes* sp. [52]. In this work, this gene was found in pathogenic *Spirochaetes* and was absent in many other genomes, suggesting its importance for the correct classification of this group. Nevertheless, *flbB* homologues were also found in *Thermoanaerobacter* (*Firmicutes*). Independently of its role in the classification of pathogens, this finding questions the evolutionary origin of this flagellar motor, apparently exclusive for *Spirochaetes*.

Bacterial motility and host-cell adhesion are intimately related processes. Fimbria (type I pili) are filamentous proteinaceous surface appendages present in many Gram-negative bacteria [53], [54] that aid the adhesion process. In *E. coli*, fimbria are made of a repeating monomer, FimA, encoded by *fimA*. This gene is almost exclusively present in pathogenic *Gammaproteobacteria* and *Betaproteobacteria*, like *Escherichia*, *Salmonella*, *Acinetobacter* and *Burkhordelia*. FimH protein (encoded by *fimH*) is the most common adhesin located on the tip of type I fimbriae [55], [56]. Its expression, hence pilus formation, is regulated by gene *fimI*, which is essential for fimbriated phenotype. Specific mutations in *fimI* lead to pilus-negative phenotype in *E. coli* and *S. enterica* [57]. Both genes, *fimH* and *fimI*, were found exactly in the same group of species belonging to *Enterobacteraceae* family: *Salmonella*, *Escherichia*, *Proteus*, *Shigella* and *Klebsiella*. This supports the functional relationship of both genes and also denotes the importance of them for classification of this family of pathogenic *Gammaproteobacteria*.

Another relevant pili aparatus is the type IV system. This macromolecular machinery is present in Gram-negative bacteria and in at least one Gram-positive [58]. Type IV pili are highly pleiotropic, being involved in bacterial motility, adhesion, immune escape, biofilm formation, secretion and phage transduction. The most relevant selected gene for this pili system was *pilA*, which codes for pilin, the major component of filament. It is present in most pathogenic *Clostridium* (*C. perfringes*, *C. tetani*, *C. difficile* and *C. botullinum*). PilA is also present in pathogenic members of a group of families belonging to *Gammaproteobacteria* (*Vibrionaceae*, *Pseudomonadaceae*, *Francisellaceae*, *Moraxellaceae*). Interestingly, *pilA* is absent in pathogenic *Enterobacteraceae*, so the combination of three genes (*pilA*, *fimH* and *fimI*) seems to explain the discrimination of most pathogenic *Gammaproteobacteria* with respect to the rest of non-pathogenic bacteria and even distinguishing between two enormous phyolgenetic groups inside this taxon.

#### Secretion systems

Several differences in secretion systems exist between Gram-positive and Gram-negative bacteria. Protein secretion across the inner membrane of both kinds of organisms generally involves the same Sec-dependent pathway, although other routes have been identified, i.e. Twin-arginine translocation (Tat) [59]–[61]. Translocation across Gram-negatives inner membrane results in release of products into the periplasmic space. Hence, these bacteria have developed several types of secretion systems which carry molecules from the periplasmic space to the cell surface or extracellular matrix. These secretory pathways of Gram-negatives can be classified into six different groups: type I to VI secretion systems (T1SS–T6SS). The presence/absence of 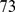 different genes coding for both shared secretory pathways (like Sec or Tat) and for T1SS–T6SS was tested. The model selected 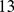 genes as the most relevant to explain class differences.

Genes for Sec system were not selected by the model. For Tat system the *tatA* gene was selected; it codes the major pore-forming subunit for translocation complex [62]. Homologues of *tatA* have been identified in a wide range of human pathogens, including *E. coli* O:157, *Vibrio cholerae*, *Mycobacterium tuberculosis*, *Listeria monocytogenes* and *Staphylococcus aureus* [63]. Moreover, this gene has orthologous in all *Epsilonproteobacteria* analyzed in this work, except for the non-pathogenic *Sulfurovum* sp. NBC37-1. Even though *tatA* was selected as an important feature for classification, a clear presence/absence pattern between pathogenic and non-pathogenic species was not observed.

Gene *yscC* encodes a key protein of the archetypical T3SS of *Yersinia pestis*, the infective agent of human plague. YscC orthologs are now identified in more than a dozen of pathogens [64], including *Salmonella enterica*, *Shigella flexneri* [65] and enteropathogenic *E. coli* [66]. Beyond these well-known examples, we identified the presence of *yscC* orthologs only in species belonging to *Gammaproteobacteria* and *Betaproteobacteria*, being absent in a great number of non-pathogenic species.

T4SS have been described in several organisms including *Bordetella pertussis* [67], *Legionella pneumophila* [68], *Brucella suis* [69], *Bartonella henselae* [59], and *Helicobacter pylori* [70]. VirB2, coded by *virB2*, is major component of T4SS pilus and has an important role in secretion [71]. Beyond its identification in the species mentioned above, *virB2* is present in some genomes of well-known pathogens with different taxonomic context: *Campylobacter jejuni* subsp. *jejuni* 81–176 (*Epsilonproteobacteria*), *Klebsiella pneumoniae* subsp. *pneumoniae* NTUH-K2044 (*Gammaproteobacteria*), *Neorickettsia sennetsu* str. Miyayama (*Alphaproteobacteria*) and three *Burkholderia* sp. species (*Betaproteobacteria*). This suggests an important role of T4SS in pathogenic processes, even in species with different pathogenic mechanisms.

T6SS have been found in species from a wide taxonomic range [72], comprising most bacterial groups included in this work. Two T6SS genes were selected: *ppkA* codes for a serine/threonine-protein kinase that phosphorylates protein FHA (encoded by *fha1*). The phosphorylation initiates a signal transduction cascade that results in T6SS assembly and function. Mutation of *P. aeruginosa fha1* gene resulted in defective secretion of Hcp1, an essential protein for pathogenesis as demonstrated by attenuated virulence phenotype observed *in vivo* [73]. Both *fha1* and *ppka* were identified in *P. fluorescens* and *P. mendocina* and all strains of *P. aeruginosa*. Interestingly, the absence of these genes in other genomes shows the great importance of their presence for the classification of these organisms exclusively. Moreover, the high correlation in the presence of both genes in the same genomes evidences their functional relationship.

### Phylogenetic Distribution of Virulence Genes

In the sections above we disscused the biological meaning of some genes selected by the model, emphasizing their presence/absence patterns among pathogens and non-pathogens and their importance in the development of pathogenic phenotypes. Here we give an integrative overview of virulence genes distribution along bacterial phylogeny, taking into account their frequency bias among pathogenic and non-pathogenic organisms. Fisher exact test (p-value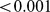) was used to select genes with significant differences in their presence/absence patterns for each functional category inside each taxonomic group. Then, gene frequency was calculated among pathogens and non-pathogens for those selected genes, separated by functional category. Finally, individual genes frequencies were added inside each group and normalized over the total number of genes belonging to each functional category.

**Figure 5:**
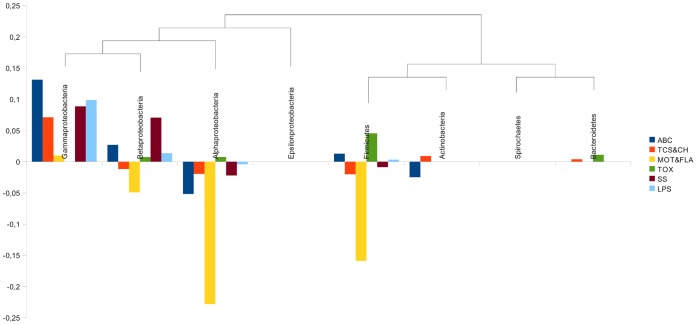
Phylogenetic distribution of virulence genes. Each functional category of virulence-related genes is represented as a vertical bar. Positive values denote association of a particular functional category with pathogenic species of a certain taxonomic group, while negative values with non-pathogenic species. Taxons are grouped according to phylogenetic relationships. In graph legend: ABC: ABC transporters, TCS&CH: two-component systems and chemotaxis, MOT&FLA: motility and flagellar assembly, TOX: toxins, SS: secretion systems, LPS: LPS biosynthesis.

Figure 5 shows normalized frequency values for genes belonging to each functional category, taking into account the phylogenetic relationships between studied taxonomic groups. Some expected patterns arise from these results, for example toxins are exclusively overrepresented in pathogenic species. This is expectable taking into account the biological purpose of toxins; it would be highly improbable that pathogenicity in a certain species was determined by the absence of a toxin that is present in the non-pathogenic species of the group. ABC transporters seem to be the most variable functional category along the phylogeny, it is positive (associated to pathogenic organisms) in *Gammaproteobacteria*, *Betaproteobacteria* and *Firmicutes*, and negative (associated to non-pathogenic organisms) in *Alphaproteobacteria* and *Actinobacteria*. This is coherent with the wide range of functions that ABC transporters can perform; for example the presence of aminoacid importers can be essential for pathogenesis of species that have lost biosynthetic genes, however, it is not contradictory with the presence of these kind of transporters in non-pathogenic species.

The most powerful association between pathogens and high gene frequencies is observed in *Gammaproteobacteria*, evidencing the importance of these kinds of genes for pathogenic species of this group, which is mainly composed of enteropathogens. The most striking result of this analysis is the pattern observed for *Alphaproteobacteria*, totally opposite to the phylogenetically related *Gammaproteobacteria*. The first question that rises is why genes previously thought of as mostly present in pathogenic species, are highly frequent in non-pathogenic species of this taxon. Marine environments contain the major component of non-pathogenic *Alphaproteobacteria* biodiversity. A recent study [74] showed that out of 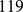 marine bacteria, 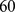 had homologues to known virulence genes from pathogenic bacteria. Interestingly, new insights in host-pathogen interactions propose a wider ecological and evolutionary perspective to better understanding the life strategy of pathogenic bacteria [75], suggesting that functions have evolved over a long time in nature and then recruited through horizontal gene transfer to perform similar or different functions in more recently emerging pathogenic species. This hypothesis opens a three-step way of thinking about how natural selection plays a role in the emergence of bacterial pathogens. First, the random appearance and fixation of new genes in bacteria colonizing inaccessible environments generate a reservoir of species carrying potentially virulent genes. Second, these bacteria can contact human hosts by movement through intermediate hosts in which they live as commensals or they can transfer virulent genes horizontally to other human-adapted bacteria. Third, positive selection over the most successful species determines the fixation of virulence genes that let bacteria to damage or survive inside human cells. The high frequency of virulence-related genes in non-pathogenic *Alphaproteobacteria* might be explained by the emergence of these kinds of genes in common ancestors for *Gammaproteobacteria* and *Alphaproteobacteria*. Then, the branch that originated *Alphaproteobacteria* conserved these genes in both pathogenic and non-pathogenic species. In contrast, *Gammaproteobacteria* could have acquired these functions by horizontal gene transfer, to produce the actual scenario of high frequency in pathogenic species and low frequency in non-pathogenic ones.

Two groups (*Spirochaetes* and *Epsilonproteobacteria*) showed very few genes with significant differences according to Fisher exact test. This reveals that for these two taxonomic groups there are no clear presence/absence patterns among genes of pathogenic and non-pathogenic species but, in spite of this, our model is able to assign each organisms to the correct class with high accuracy. This is particularly interesting because our model is using information coded in high-dimensional spaces, leaving behind the simple presence/absence patterns. Moreover, here we could identify only some particular associations between phylogeny topology and functional categories, suggesting that, in general, the functional importance of these genes varies along bacterial taxonomy. The lack of general patterns between the presence of functional categories and phylogenetically related groups supports the notion that most virulence-related genes are spread among bacteria by horizontal gene transfer. Probably our method is taking benefit of this scenario, being able to correctly classify organisms independently of their taxonomic context, based on widely spread genes along bacterial phylogeny.

### Misclassified Organisms

A group of 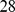 out of the 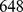 genomes tested were systematically misclassified by the model. We defined a genome to be misclassified if it was assigned to the wrong class, at least in 50% of 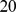 consecutive classifications (Table S4). Ten out of these 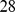 are labeled as human pathogens but the model returned them as non-pathogenic, while 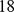 out of 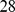 are labeled as non-pathogenic but were classified as human pathogens. Most cases of misclassification are observed in species with a big number of sequenced genomes of different strains. This is the case of *Staphylococcus aureus*, an important human pathogen. Thirteen out of the 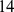 genomes of different strains of this species were well classified as human pathogens. Nevertheless, the strain *S. aureus* subsp. *aureus* MRSA252 was assigned to the non-pathogenic class. Comparison of present/absent genes for all *S. aureus* genomes showed that gene *hlyII* (coding for hemolysin II) was absent in *S. aureus* subsp. *aureus* MRSA252 while present in the rest. This was the only difference between these genomes; moreover gene *hlyII* was one of the 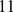 toxin-coding genes selected as more informative during the feature selection process. On the one hand, this fact shows that for a particular species even the presence of a single feature is determining the classification of the genome as pathogenic or non-pathogenic, indicating a great power of some genes in determining the class assignment by the model. On the other hand, it is possible to misclassify genomes due to a particular gene loss, especially in those cases of high genetic variability among strains of certain species.

For misclassified genomes that do not have other well-classified strains belonging to the same species, it is not possible to assess the present/absent comparison to find differences in gene patterns. In these cases, misclassification can be explained by inherent errors of SVM model construction or because the features (groups of orthologous genes) originally used to determine the presence/absence matrix, might not be informative enough to reach a 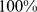 classification performance. However, in some cases it is possible to propose a biological explanation for misclassification, based on the particular ecological and genetic features of some species.

The first example is *Bordetella petrii* (*Betaproteobacteria*) which is originally labeled as non-pathogenic, but the model classifies it as pathogenic. This could be primarily seen as a classification error, but there is strong evidence that supports this species is an emerging human pathogen. Though being an environmental isolate, the sequenced *B. petrii* DSM12804 strain also encodes proteins related to virulence factors of the pathogenic *Bordetellae*, including the filamentous hemagglutinin, which is a major colonization factor of *B. pertussis*. The genomic analysis of *B. petrii* suggests an evolutionary link between free-living environmental bacteria and the host-restricted obligate pathogenic *Bordetellae* [76]. Moreover, clinical isolates of *B. petrii* have been recently described to cause, for example, mandibular osteomyelitis [77] or supurative mastoiditis [78].

Other example comprises a group of 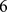 marine non-pathogenic *Alphaproteobacteria* (*Rhodobacter capsulatus*, *Erythrobacter litoralis*, *Rhodopseudomonas palustris*, *Novosphingobium aromaticivorans*, *Parvularcula bermudensis* and *Sphingobium japonicum*), wrongly classified as pathogenic. As explained in the section above, *Alphaproteobacteria* have the highest frequency of virulence-related genes in non-pathogenic species. The 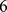 misclassified species shared the presence of 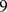 genes involved in secretion processes, supporting the findings of Persson et al. [74] regarding the extensive appearence of these kinds of genes in marine bacteria. Despite this, only 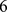 out of 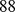 *Alphaproteobacteria* were misclassified, indicating that the classification model can deal with unexpectedly biased gene frequencies towards non-pathogenic organisms without compromising classification performance.

### Model Sensitivity

A simple approach to evaluate the sensitivity of the constructed model is to assess the propensity of label shift (pathogens to non-pathogens and vice versa). This experiment was implemented for each taxonomic group in the dataset by artificially modifying presence/absence vectors. For each genome those present genes were systematically “turned off” one at a time, running the classification model each time and recording in which cases a category shift occurred. The same strategy was used to “turn on” those genes which were originally absent.

The change from non-pathogen to pathogen was lead by a group of 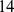 genes, which were mainly toxin-coding genes (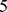) and TCS (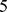). These two functional categories together comprise 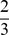 of the genes that influence the category shifting in the mentioned direction, evidencing a great importance of these features as exclusive determinants of bacterial pathogenicity. Individually, the presence of any of these genes is able to change a number of organisms ranging from 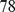 to 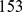, depending on the gene. The most extreme is the case of SLO toxin, whose presence determines that 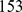 species change from non-pathogens to pathogens.

Changing from pathogen to non-pathogen is mainly determined by gene “turn off”. A group of 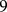 genes are responsible for category shifting in this direction, changing the classification of 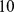 to 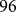 species. It is worth mentioning that the gene coding for the SLO toxin is one of the most influential; this makes sense, since the gain of this gene provoked a label change to pathogen, it is expectable that losing it defines a label change to non-pathogen.

### Software Development: The BacFier

BacFier v1.0 was implemented as a Java software, and hence platform independent, in order to make it easier for the common user to work with the model. A simple interface allows the user to upload the genome sequence (finished or unfinished) of the organism of interest. The genome is used as query to perform BLAST against the final set of 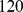 orthologous groups (selected as explained in section Model construction) creating a presence/absence vector for the genome. The vector is evaluated with a SVM model, and an outcome (pathogen/non-pathogen) is produced associated to a probability.

Moreover, the sensitivity analysis described in the previous section can be automatically performed with the software, this is assessed by selectively “turning off” or “turning on” desired genes in the presence/absence vector and re classifying the result. This might indicate genes that are likely to change the label of the organism, so that one can pay more attention to them and corroborate their status of presence/absence. Furthermore, this strategy becomes crucial when inputing an unfinished genome. In this situation, the absence of some genes important for pathogenicity could be determined by the unfinished status of the genome, so if prediction result is non-pathogenic, the user can sistematically “turn on” those absent genes until the model shift to pathogenic. Then, the real presence of genes that determined the shift can be investigated by a more refined search or by other methods, like PCR.

BacFier v1.0 is freely available under 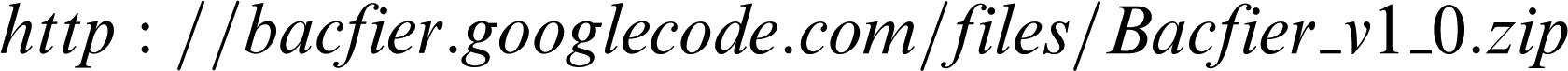.

### Conclusions

The constructed SVM model classifies bacterial genomes in human pathogens and non pathogens with 95.4% of average accuracy. To the best of our knowledge, this is the statistical model with this purpose that achieves the highest accuracy reported so far. Moreover, our method classifies bacterial genomes independently of their taxonomic context, in contrast to other similar approaches that only take into account a certain part of bacterial diversity, being useful only to classify specific taxa [6]. Our statistical learning approach is grounded on the biological meaning of the selected genes and supported by the fact that bacterial pathogenicity can be explained by the presence or absence of a set of specific genes that code for virulence determinants. The application of BacFier v1.0 may be useful for clinical or industrial purposes, for example to determine if a new sequenced strain could be pathogenic for humans.

## Methods

### Data Selection and Matrix Construction

Complete genome sequences from all available bacteria were downloaded from the National Center for Biotechnology Information (NCBI). Over 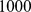 genomes were obtained and from those organisms, we originally kept 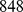 that were labeled as human pathogens or non-pathogens. This set of bacteria comprehends 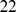 taxonomic groups. In this work, we focused only on human pathogens; if a certain species was a multi-host pathogen including humans, it was considered human pathogen. By the contrary, if a certain species was a multi-host pathogen or a pathogen of other host different from human, it was excluded from the dataset considered.

Eight gene functional categories that we considered related to pathogenicity were determined. These are toxins, chemotaxis proteins, ABC transporters, motility proteins, LPS biosynthesis, two-component systems, flagellar assembly and secretion systems. Orthologous groups from proteins coded by genes belonging to these categories were downloaded from KEGG Orthology database (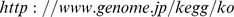), all the categories together resulted in 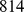 orthologous groups. With this data, we built a presence/absence table showing which orthologous groups (genes/proteins) were present or absent in the organisms considered. We selected local protein BLAST [79] searches to perform orthologous genes determination. Not only does this approach absolve us from using a refined orthologous search method (which can be much more laborious and time-consuming), but it also provides good enough accuracy in orthologous determination. In this case, our method must be robust and tolerant enough to identify possible false positive or false negative orthologs.

BLAST searches were performed formatting the 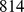 orthologous groups and querying the organisms. If an alignment between an organism and a gene (member of an orthologous group) was “good enough” (see below), then we considered the gene (orthologous group) as present in the organism, otherwise as absent. This, is represented as a 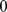/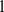-matrix with dimensions 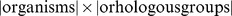. We defined “good” alignments as the ones having a percentage of identity higher than 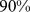, length of the alignment larger than 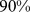 of the gene’s length and an e-value smaller than 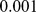. Further analyses were made on 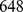 genomes belonging to 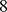 of the 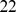 taxonomic groups: *Actinobacteria*, *Alphaproteobacteria*, *Bacteroidetes*/*Chlorobi*, *Betaproteobacteria*, *Epsilonproteobacteria*, *Firimicutes*, *Gammaproteobacteria* and *Spirochaetes*, since there were not enough genomes available for the other groups. However, these excluded genomes were then used as part of external groups to further test the constructed model.

### Model Construction

In this work a machine learning approach based on a cross-fold validation with in-fold feature selection was developed. This technique ensures that particular predictions are not biased by overselected features or overfitting since each prediction is performed without using the sample in neither the feature selection nor the classifier building process. Algorithm 1 shows the methodology.

**Table:** 

Algorithm 1. General overview of cross-fold validation with in-fold feature selection (be X = whole set of samples).
**for ** 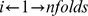 **do**
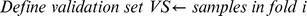
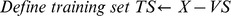
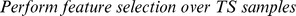
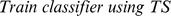
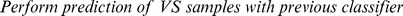
**end for**

The number of folds (nfold) was set to 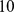 and the feature selection routine was SVMAttributeEval from Weka [80]. Regarding the classification algorithm, a Support Vector Machine (SVM) was employed. The SVM method performs the classification by constructing an N-dimensional hyperplane that optimally separates the data into two classes. In this case classes are labeled as human pathogens and non-pathogens. The raw dataset of variables is defined by the presence/absence of orthologous groups in the genomes of the organisms considered. It is important to note that the taxonomy is not used as another variable in the model since it would introduce an artificial separation in the SVM model training.

Following the spirit of Occam’s razor, in this work a linear SVM model is proposed. Although the number of genes looks relatively large, it is worth to mention that the model variables encode low level information related to gene presence/absence in each organism. Also, it is well known that linear SVM models benefit from using these kinds of variables since higher dimensions allow easier class separation. The subroutine libsvm in Weka was also employed [80].

A final analysis was done in order to determine an appropiate number of features to retain. Experiments were carried out considering 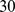, 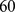, 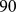, 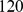, 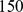, 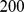 and 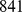 (entire set of genes) features. The accuracy obtained in each case was 90%, 93.5%, 94.4%, 95.4%, 95.5%, 94.9% and 92.1% respectively. A set of 120 genes was then considered, as they represent a reasonable tradeoff between accuracy prediction and the number of genes used for prediction.

From Algorithm 1 is clear that a different set of features can be selected in each loop of the cross-validation procedure. However, it is necessary to find a final set of genes to build a classification model and check and external validation set (for practical purpose) or predict pathogenicity of new sequenced bacteria. A common solution is to employ a voting scheme that sums how many times a feature is selected in each loop of Algorithm 1. In this particular case, the list of genes selected is available in Table S1.

#### Y-randomization test

Since in this work a binary occurrence matrix is used to represent the presence/absence of genes in a set of organisms, the number of calculated variables is high, as expected. In this particular case, the number of genes is 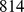. A feature selection technique further reduced the set to the 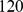 most significant variables. Although this meets the rule of thumb that states the ratio between number of samples (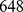 organisms) and variables (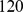) must be greater than 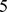 [81], problems associated with chance correlation could still arise. This is a major concern when the prediction model is expected to be reliable in terms of generalizability.

The y-randomization validation method tries to observe the influence of chance when fitting any given data. This is done by deliberately destroying the relationship between the target y and the independent variables x (genes, in this case). This is done by randomly shuffling the y data, preserving all x data untouched, and retraining the learning algorithm. A common pitfall is to apply the y-randomization procedure but using the same set of variables resulting from the feature selection process. Following the good-practice procedures, in this work the test was carried out using the full set of variables, so there was no “overestimation” (in the sense of chance correlation).

In this work we have two classes, so the expected behavior was to obtain an accuracy of roughly 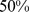 in the y-randomization test (since 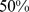 is the probability of a “good” prediction when no relation is found between variables and targets, the same as a random assignment of predicted labels). In this work the y-randomization procedure was carried out 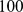 times (Figure S1).

### Genes Significance and Frequency Calculation

In order to weight the importance of each functional category for each taxonomic group, we selected those genes with statistically significant presence/absence patterns inside pathogens and non-pathogens. Fisher exact test was applied to genes belonging to each functional category for each taxonomic group. Those genes with p-value 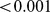 were taken into account. Then, the frequency of those genes was calculated for pathogenic and non-pathogenic species of each taxonomic group, as the number of presences over the total number of organisms inside the group. Finally, for a certain functional category, the significance value was calculated as the accumulated frequency of those genes significant to the category, and normalized over the total number of genes belonging to it. For a better graphical visualization of Figure 5, frequencies in non-pathogenic organisms were multiplied by 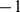, in this way positive values are associated with pathogenic organisms while negative with non-pathogenic ones.

## Supporting Information

Figure S1 Y-randomization performance over 100 runs.
(TIF)

Table S1 Description of the subset of 120 selected genes.
(XLS)

Table S2 Classification results for each tested genome in 10-fold cross validation.
(XLS)

Table S3 Prediction for each organism belonging to test Group II. These organisms were previously subjected to a bibliography revision to determine their assignation to human pathogens or non-pathogens. When organism resulted to be pathogen, citation is reported. Column Prediction shows the result after model prediction, correctly classified organisms are highlighted in green while wrongly classified are in red.
(XLS)

Table S4 List of misclassified organisms.
(XLS)
